# Effective inhibition of HCMV UL49 gene expression and viral replication by oligonucleotide external guide sequences and RNase P

**DOI:** 10.1186/1743-422X-7-100

**Published:** 2010-05-18

**Authors:** WenJun Zhang, HongJian Li, YueQin Li, ZhiFeng Zeng, ShiQian Li, Xin Zhang, Yi Zou, TianHong Zhou

**Affiliations:** 1 National Engineering Research Center of Genetic Medicine, Jinan University, Guangzhou, 510632, China; 2 Department of Pathogen Biology and Immunology, Guangdong Pharmaceutical University, Guangzhou, 510006, China

## Abstract

**Background:**

Human cytomegalovirus (HCMV) is a ubiquitous herpesvirus that typically causes asymptomatic infections in healthy individuals but may lead to serious complications in newborns and immunodeficient individuals. The emergence of drug-resistant strains of HCMV has posed a need for the development of new drugs and treatment strategies. Antisense molecules are promising gene-targeting agents for specific regulation of gene expression. External guide sequences (EGSs) are oligonucleotides that consist of a sequence complementary to a target mRNA and recruit intracellular RNase P for specific degradation of the target RNA. The UL49-deletion BAC of HCMV was significantly defective in growth in human foreskin fibroblasts. Therefore, UL49 gene may serve as a potential target for novel drug development to combat HCMV infection. In this study, DNA-based EGS molecules were synthesized to target the UL49 mRNA of human cytomegalovirus (HCMV).

**Results:**

By cleavage activity assessing *in vitro*, the EGS aimed to the cleavage site 324 nt downstream from the translational initiation codon of UL49 mRNA (i.e. EGS324) was confirmed be efficient to direct human RNase P to cleave the target mRNA sequence. When EGS324 was exogenously administered into HCMV-infected human foreskin fibroblasts (HFFs), a significant reduction of ~76% in the mRNA and ~80% in the protein expression of UL49 gene, comparing with the cells transfected with control EGSs. Furthermore, a reduction of about 330-fold in HCMV growth were observed in HCMV-infected HFFs treated with the EGS.

**Conclusions:**

These results indicated that UL49 gene was essential for replication of HCMV. Moreover, our study provides evidence that exogenous administration of a DNA-based EGS can be used as a potential therapeutic approach for inhibiting gene expression and replication of a human virus.

## Background

Human cytomegalovirus is a ubiquitous herpesvirus that typically causes asymptomatic infections in healthy individuals but may lead to serious complications in newborns and immunodeficient individuals. For example, this virus accounts for one of the most common opportunistic infections in AIDS patients (i.e., CMV retinitis). Moreover, HCMV infection is the leading viral cause of birth defects in newborns and a major cause of morbidity and mortality in bone marrow and solid organ transplant recipients [1]. The emergence of drug-resistant strains of HCMV has posed a need for the development of new drugs and treatment strategies [2].

**Figure 1:**
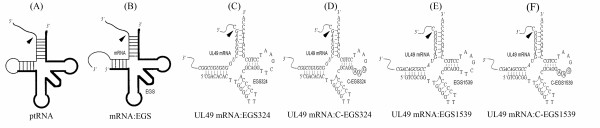
**Schematic representation of substrates for RNase P**. (*A*) A natural substrate (ptRNA). (*B*) A hybridized complex of a target RNA (e.g., mRNA) and an EGS that resembles the structure of a tRNA. (*C*-*F*) Complexes between UL49 mRNA sequence and EGS324, C-EGS324, EGS1539, and C-EGS1539 respectively. The sequence of these EGSs equivalent to the tRNA sequence was derived from tRNA^Ser ^and resembles the T-stem and loop, and variable region of the tRNA molecule.

Antisense molecules are promising gene-targeting agents for specific regulation of gene expression [3]. Conventional antisense oligonucleotides have been used as anti-HCMV agents to inhibit the expression of HCMV-essential genes and abolished viral replication [4,5]. External guide sequences (EGSs) are antisense oligonucleotides that can be used in conjunction with ribonuclease P (RNase P) or the catalytic RNA subunit of RNase P from *Escherichia coli *(M1 RNA) to diminish gene expression [6-9]. RNase P is one of the most abundant and active enzymes in cells and is responsible for 5' termini maturation of tRNAs [10]. This enzyme catalyzes a hydrolysis reaction to remove the 5' leader sequence of tRNA precursors (ptRNA) by recognizing the common structure shared among all tRNAs (Fig. 1A). The EGS-based technology is unique in inducing endogenous RNase P to cleave a target mRNA when the EGS hybridizes to the mRNA to form a structure resembling a ptRNA substrate (Fig. 1B). This approach is highly specific and does not generate nonspecific "irrelevant cleavage" that is observed in RNase H-mediated cleavage induced by conventional antisense phosphothioate molecules [11]. Thus, EGSs represent a new class of agents that may lead to highly effective and specific inhibition of gene expression.

RNA-based EGSs have been expressed endogenously as transgenes in both bacteria and mammalian cells [12], and were effective in inhibiting the gene expression of herpes simplex and influenza virus and in abolishing the replication of influenza virus in human cells [13,14]. *In vitro *studies have also shown that DNA-based EGSs, as well as EGS molecules with modified nucleotides, can direct M1 RNA or human RNase P to cleave a mRNA sequence, although their targeting efficiencies are lower than those of unmodified RNA-based EGSs [15]. However, little is known about whether DNA-based EGSs are functionally active in cultured cells. Also, whether DNA-based EGSs can be exogenously administered into human cells to inhibit gene expression and growth of human viruses is yet to be determined.

The UL49-deletion BAC of HCMV was significantly defective in growth in human foreskin fibroblasts [16]. Furthermore, the expression of HCMV UL49 protein has been shown in our mock infected human foreskin fibroblasts (unpublished data, doctoral dissertation). Therefore, UL49 gene may serve as a potential target for novel drug development to combat HCMV infection. In this study, we provide direct evidence that exogenous administration of chemically synthesized DNA-based EGS is highly effective in inhibiting HCMV gene expression and growth in human cell culture and, furthermore, demonstrate the feasibility of using DNA-based EGSs for the studies and treatment of infections caused by human viruses including HCMV.

## Materials and methods

### Construction of the RNA substrate and EGSs

The DNA sequence that encodes HCMV UL49 gene was constructed by PCR using AD169 genomic DNA as a template and oligonucleotides 49F: 5'-AGTTGGATCCATGGCCAGTCGTCGTCTC-3' and 49R: 5'-ATCTAAGCTTGTAGACATGGGGCAGGCCGT-3' as 5' and 3' primers, respectively. The PCR fragments were digested with BamH I and Hind III, and then inserted into vector pGEM3z (Invitrogen) to form a recombinant plasmid, *UL49-pGEM3z*. The RNA substrates were synthesized by bacteriophage T7 RNA polymerase using the Hind III-linearized *UL49-pGEM3z *plasmid as transcriptional templates and labeled with ^32^P-UTP. The restriction endonucleases, LA Taq polymerase, T4 DNA ligase and T7 RNA polymerase were purchased from Takara (Dalian, China). All oligonucleotides used as PCR primers were purchased from Invitrogen (Guangzhou, China). EGS oligonucleotides used in this report including EGS324(5' CGACACACTTGGGGTTTCCCCACGCAGGTTCGAATCCTGCGCCATTCCCA3'), EGS1539(5'GTCGCGGTTGGGGTTTCCCCACGCAGGTTCGAATCCTGCCTCGCCCCCA3'), C-EGS324(5' CGACACACTTGGGGTTTCCCCACGCAGGAAGGAATCCTGCGCCATTCCCA3'), C-EGS1539(5'GTCGCGGTTGGGGTTTCCCCACGCAGGAAGGAATCCTGCCTCGCCCCCA3'), and TK-EGS(5'TACGTCGGTGCGGTCTCCGCGCGCAGGTTCAAATCCTGCCGCAGACACCA3') were chemically synthesized directly by using a DNA synthesizer.

### Purification of human RNase P

RNase P was purified from HeLa cells according to the method as described [17] with the following modifications. The DEAE-Sepharose Fast-flow column was equilibrated and washed with buffer A containing 10 mM Tris-HCl, pH 7.5, 2.5 mM MgCl_2_, 5 mM KCl, 2 mM DTT, and 1 mM Pefabloc (proteinase inhibitor). The enzyme was eluted with a linear gradient of 100~500 mM KCl in the same buffer. The active fractions were pooled and concentrated to a final volume of approximately 5 ml. The active fractions were pooled and dialyzed against a buffer containing 10 mM Tris-HCl, pH 7.5, 2.5 mM MgCl_2_, 5 mM KCl, 2 mM DTT, 1 mM Pefabloc and 50% glycerol. The samples were then stored at -80°C before use. The specific activity of the RNase P was determined as the cleavage of 0.02 pmol yeast tRNA^ser ^precursor per min per μl.

### *In vitro *cleavage studies

The EGSs (50 nM) and ^32^P-labeled UL49 mRNA (50 nM) were incubated with human RNase P (5 units). The cleavage reactions were carried out at 37°C for 30 min in buffer A (50 mM Tris-HCl, pH7.5, 100 mM NH_4_Cl, 10 mM MgCl_2_). To disrupt aggregates, which might form during storage, EGS and substrate together were heated to 90°C for 1 min and then cooled to room temperature before the addition of other components. Substrate and cleavage products were separated under denaturing conditions on a 15% acrylamide gel containing 8 M urea. The amount of radioactivity per band was quantified using a *Typhoon 9200 *phosphorimager. The procedures to measure the equilibrium dissociation constants (*K*_*d*_) of the EGS-UL49 mRNA complexes were carried out as described [18]. In brief, various concentrations of EGSs were preincubated in buffer B (50 mM Tris, pH 7.5, 100 mM NH_4_Cl, 20 mM MgCl_2_, 3% glycerol, 0.1% xylene cyanol, 0.1% bromphenol blue) at 37°C for 10 min before mixing with an equal volume of different concentrations of ^32^P-labeled substrate RNA preheated under identical conditions. The samples were incubated for 15 min to allow binding, loaded on a 5% polyacrylamide gel, and run at 10 watts. The electrophoresis running buffer contained 100 mM Tris-Hepes, pH 7.5, and 10 mM MgCl_2_. Each band was quantitated with a Typhoon 9200 phosphorimager. The value of *Kd *was then extrapolated from a graph plotting percentage of product bound *versus *EGS concentration. The values were the average of three experiments.

### EGS internalization and viral infection

Lipofectamine 2000 reagent (GIBCO) was diluted in 100 μl of Opti-MEM medium with the EGS to give a final concentration of 10 ug/ml lipid-100 nM EGS. The transfection experiments were carried out by using 100 nM EGS. The EGS-lipid mixtures were prepared according to the manufacturer's recommendation (GIBCO) and incubated with cells for 7 hrs. HCMV (strain AD169) was propagated in human foreskin fibroblasts in DMEM supplemented with 10% FBS (GIBCO). Cells (1 × 10^6^) were treated with liposome complexes in the absence and presence of EGS, followed by HCMV infection or mock infection at a MOI of 1 in an inoculum of 1.5 ml DMEM supplemented with 1% fetal calf serum (FCS). Total cellular RNAs and proteins were isolated from cells as described 72 hrs postinfection [19].

### Fluorescence Quantitative RT-PCR

Fluorescence Quantitative RT-PCR (FQ-RT-PCR) was performed using standard protocols on an Applied Biosystem's 7300 Sequence Detection System. Briefly, total RNAs were extracted using Trizol, then 5 ml of a 1/100 dilution of cDNA in water was added into 12.5 ml of the 2 × SYBR green PCR master mix (Takara), with 800 nM of each primer in a total volume of 25 ml. All reactions were run in triplicate using Applied Biosystem's 7300 Sequence Detection System. UL49 gene sense primer: 5'-CGTTCTTGCGTCCTTCATCT-3'; anti-sense primer: 5'-CACAAAGTAGGGCTTGGTCAT-3'. As internal standard, a fragment of human endogenous β actin was amplified simultaneously in each PCR. β actin sense primer: 5'-TCGTCCACCGCAAATGCTTCTAG-3', anti-sense primer: 5'-ACTGCTGTCACCTTCACCGTTCC-3'. Quantitative RT-PCR conditions: 95°C for 10 s, 1 cycle, followed by 95°C for 5 s, 60°C for 20 s, 40 cycles for amplifying UL49 and β actin.

### Western blotting

For Western analyses, the denatured, solubilized polypeptides were separated on 12% (vol/vol) SDS polyacrylamide gels cross-linked with N, N"-methylenebisacylamide, and then transferred onto PVDF membranes (Amersham, Freiburg, Germany). The membranes were incubated in the primary antibody solution (including antibody against human β actin, ul49, IE1/IE2, pp28, or gB), then were incubated in a goat anti-mouse HRP-conjugated antibody solution. After washing with PBS containing 0.05% Tween 20, the membranes were subsequently stained with a chemiluminescent substrate with the aid of a Western chemiluminescent substrate kit (Pierce, Rockford) and quantitated with a *Typhoon 9200 *phosphorimager [20]. The polyclonal antibodies against HCMV UL49 protein were kindly provided by Doctor Feng Zhu (BOAOSEN, Beijing). The monoclonal antibodies, which react with HCMV proteins gB, pp28 and IE1/IE2, respectively, were purchased from SANTA CRUS. The monoclonal antibodies against human β actin was purchased from SIGMA.

### Assay of viral growth in EGS-treated cells

To determine the level of the inhibition of viral growth, 5 × 10^5 ^human foreskin fibroblasts were first incubated with liposome complexes in the absence and presence of EGSs, and then mock-infected or infected with HCMV AD169 at a MOI of 1. The cells and medium were harvested at 1, 2, 3, 4, 5, 6, and 7 days postinfection. The viral stocks were prepared by adding an equal volume of 10% skim milk, followed by sonication. The titers of the viral stocks were determined by infecting 2 × 10^5 ^foreskin fibroblasts in 6-well plates and counting the number of plaques 7~10 days postinfection. The values obtained were the average from triplicate experiments.

## Results

### Design of EGSs

To achieve optimal cleavage, it is critical to choose the target regions of the UL49 mRNA. According to general principles of designing EGSs [21], two positions, 324 nt and 1539nt downstream from the translational initiation codon, were chosen as the cleavage sites for EGSs. The flanking sequence of these cleavage sites exhibited several sequence features that need to be present in order to interact with an EGS and RNase P to achieve efficient cleavage. These features include that ➀ the nucleotides 3' and 5' adjacent to the site of cleavage are a guanosine and a pyrimidine, respectively, and ➁ a U is 8 nt downstream from this cleavage site. The interactions of these sequence elements with the EGS facilitate the formation of the mRNA-EGS complex into a ptRNA-like structure while those with RNase P are critical for recognition and cleavage by the enzyme [22]. On the basis of the target sites, two EGSs (EGS324 and EGS1539) were constructed respectively, which resemble a part of the tRNA^Ser ^structure, containing a T-loop, a stem, and a variable region but not the anticodon region (Fig. 1C and 1E). The anticodon domain has been shown to be dispensable for EGS activity. Binding of EGSs to the target mRNA results in a helix structure of 7 bp that is equivalent to the D stem of a tRNA, which usually has 4 bp. Our choice of a D stem-equivalent structure of 7 bp instead of 4 bp is to increase the targeting sequence specificity of the EGS (Fig. 1C and 1E). The two control (C-EGS324 and C-EGS1539) were derived respectively from EGS324 and EGS1539 by introducing base substitution mutations in three positions of the T-loop (Fig. 1D and 1F). The nucleotides in these three positions are highly conserved among tRNA molecules and are important for the recognition of tRNA molecules by RNase P [23].

### *In vitro *studies of targeting activities of the EGSs

**Figure 2:**
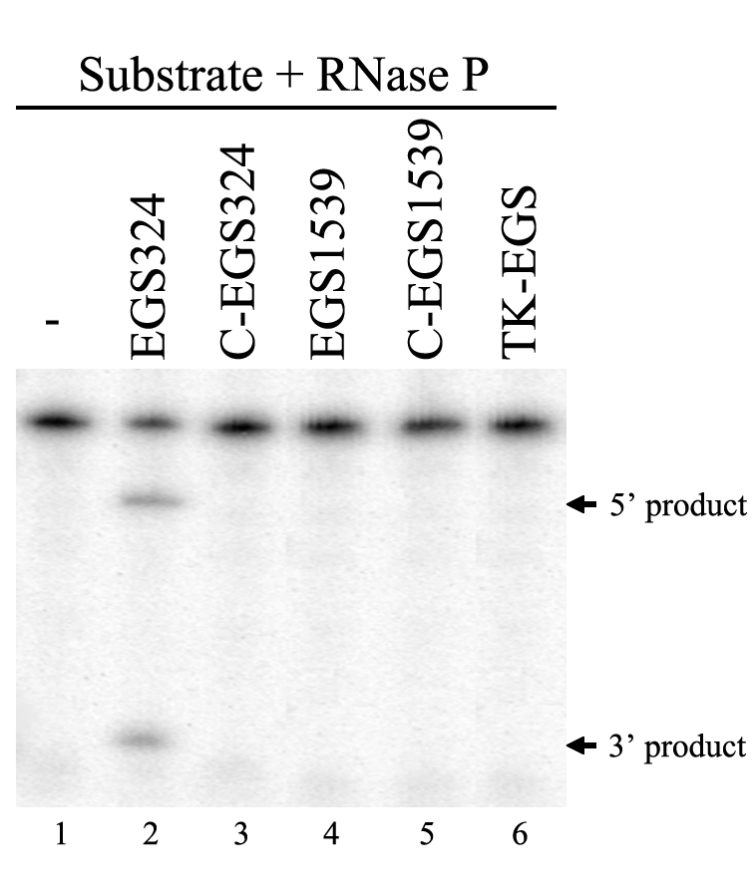
**Cleavage of the ^32 ^P-labeled substrate by human RNase P in the presence of different EGSs**. No EGS was added to the reaction mixture in lane 1. Cleavage reactions were carried out in the presence of EGS324 (lane 2), C-EGS324 (lane 3), EGS1539 (lane 4), C-EGS1539 (lane 5) or TK-EGS (lane 6).

**Figure 3:**
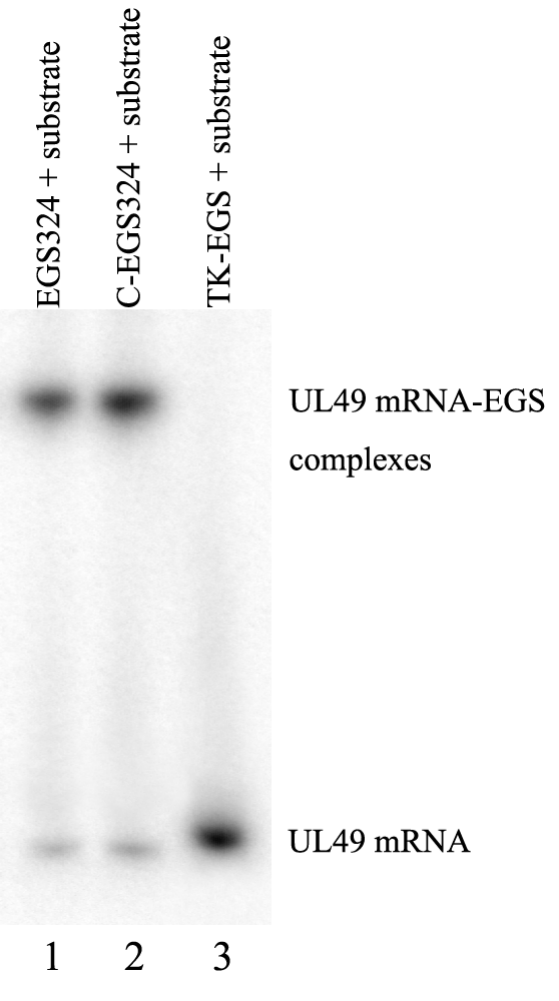
**Binding of UL49 mRNA substrate by EGS324 and C-EGS324**. Substrate (10 nM) was either in the presence of 10 nM of EGS324 (lane 1), C-EGS324 (lane 2), or TK-EGS (lane 3) to allow binding and then loaded on a 5% polyacrylamide gel.

The EGSs were subsequently incubated with human RNase P and substrate RNA, containing the targeted UL49 mRNA sequence of 1713 nt. In the absence of any EGS, no cleavage of the UL49 mRNA sequence was observed (Fig. 2, lane 1). Efficient cleavage of this substrate by RNase P was observed in the presence of EGS324 (Fig. 2, lane 2). In contrast, cleavage of the same substrate by RNase P was barely detected in the presence of C-EGS324 (Fig. 2, lane 3). It is possible that the differential cleavage efficiencies observed with EGS324 and C-EGS324 were due to their different binding affinities to the UL49 mRNA sequence. To investigate whether this was the case, the binding between the EGSs and substrate UL49 was studied using a gel-shift assay. In this assay, the EGSs were incubated with the substrate to allow for binding, and the EGS-UL49 mRNA complexes were separated in polyacrylamide gels under non-denaturing conditions. Similar amounts of complexes formed between these two EGSs and the UL49 mRNA sequence were observed when the same amount of EGSs was used (Fig. 3, lane1 & lane 2). Further detailed assays under different concentrations of the EGS324 and UL49 mRNA indicated that the binding affinity of C-EGS324 to UL49 mRNA (*K*_*d *_= 0.83 ± 0.12 μM) is similar to that of EGS324 (*K*_*d *_= 0.82 ± 0.10 μM). Meanwhile, a very little amount of cleavage products was observed in the presence of C-EGS324 even under high concentrations of the EGS and RNase P and a prolonged incubation period (data not shown). These observations suggest that the mutations in C-EGS324 do not significantly affect the binding affinity of the EGS to the mRNA sequence but abolish its targeting activity to induce RNase P cleavage, possibly by disrupting the recognition of EGS-UL49 mRNA complex by RNase P. Thus, C-EGS324 may be used as a control for the antisense effect in our experiments in cultured cells (see below). Nevertheless, both EGS1539 and C-EGS1539 showed no activity in guiding RNase P to cleave the target RNA (Fig. 2, lane 4 & lane 5). An additional EGS, TK-EGS, which was targeting the HSV-1 TK mRNA, was also constructed. This EGS was used to determine whether an EGS with an incorrect targeting sequence can direct human RNase P to cleave UL49 mRNA. No cleavage of UL49 mRNA by RNase P was observed *in vitro *in the presence of TK-EGS (Fig. 2, lane 6).

### Inhibition of HCMV UL49 expression in EGS-treated cells

**Figure 4:**
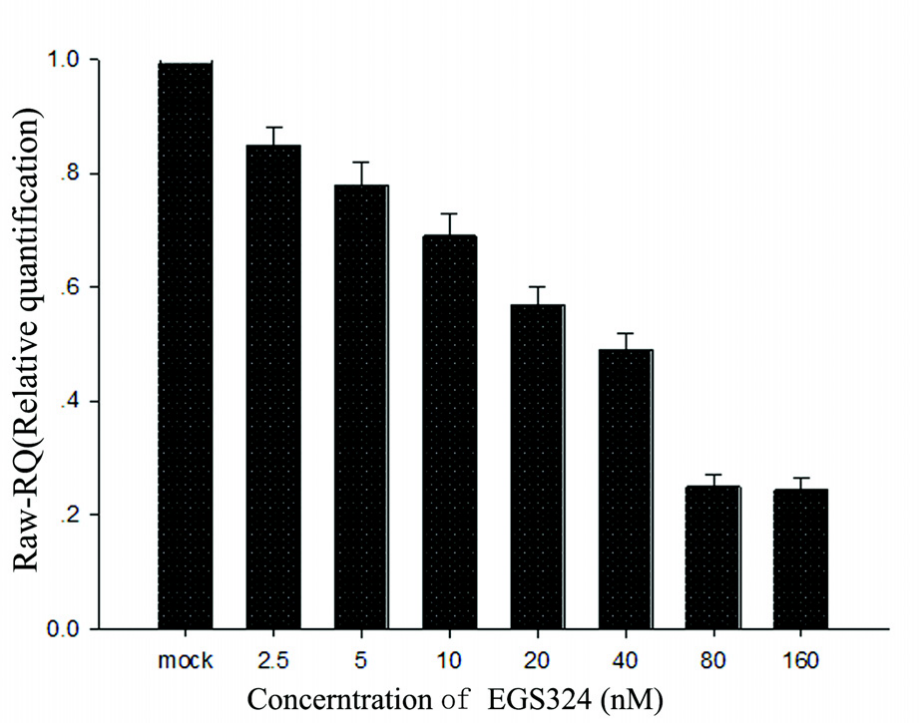
**Inhibition of HCMV UL49 expression in EGS-treated cells**. HFFs (10^6^) that were first treated with liposome complexes in the absence and presence of EGSs (100 nm) were either mock-infected or infected with HCMV at a moi of 1. At 72 hrs postinfection, total cellular RNAs were isolated, and then analyzed by quantative RT-PCR. The values shown are the means from triplicate experiments.

The chemically synthesized EGS molecules were complexed with Lipofectamine 2000 liposomes and delivered into human foreskin fibroblasts. Treatment of cells with the EGS-liposome complexes by using our transfection protocol consistently yielded a transfection efficiency of about 90% (data not shown). To investigate whether the internalized EGSs inhibit viral UL49 expression, the cells were treated with EGSs and then infected with HCMV at a moi of 1. The levels of viral UL49 mRNA were determined by quantitative RT-PCR analyses (Fig. 4). A reduction of 75.57 ± 3% in the level of UL49 mRNA expression was observed in cells treated with EGS324, whereas a reduction of less than 10% was observed in the cells treated with C-EGS324. No reduction in the expression level of UL49 mRNA was observed in cells that were treated with liposome complexes in the absence of EGSs or in the presence of TK-EGS. These results suggest that the significant reduction of UL49 mRNA expression in the EGS324-treated cells was due to targeted cleavage by RNase P. The low level of inhibition observed in the C-EGS324 treated cells was presumably due to the antisense effects of the EGS.

**Figure 5:**
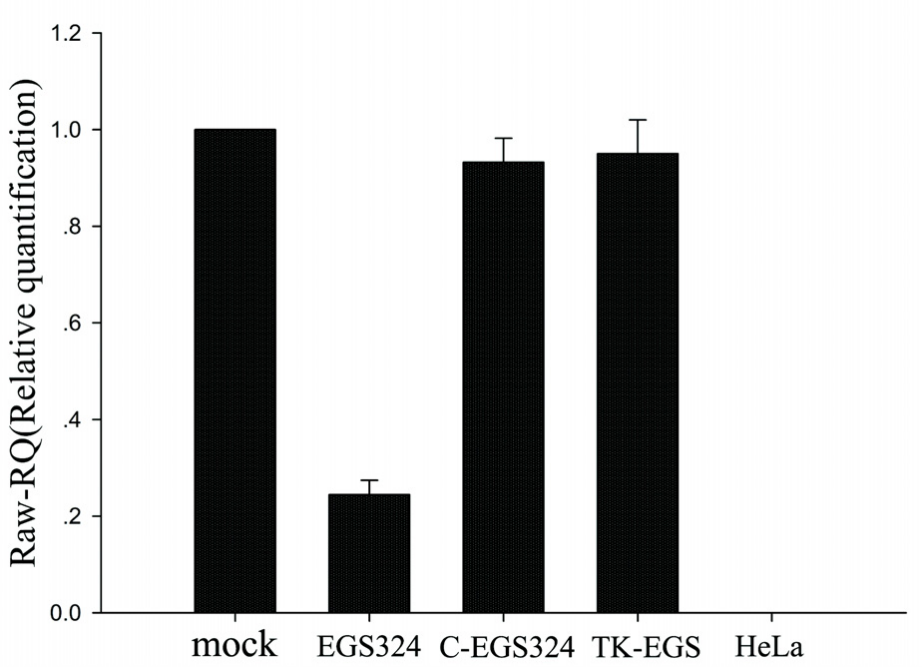
**Inhibition directed by DNA-based EGS is dose-dependent**. Quantitative RT-PCR showed that when the concentration of EGS324 was 2.5 nM, the inhibition efficiency was 15%, and then 5 nM was 22%, 10 nM was 31%, 20 nM was 43%, 40 nM was 51%, 80 nM was 75%, and 160 nM was 75.6%. Mock group was treated with a 50 bp random DNA fragment.

To determine whether the inhibition of UL49 expression directed by EGS324 is dose-depended, the EGS324 were transfected into HFFs at different concentrations (2.5, 5, 10, 20, 40, 80, and 160 nM), followed by infection with HCMV at a moi of 1. At 72 hrs postinfection, total RNAs were extracted using Trizol reagent, and then quantitative RT-PCR were performed. The quantitative RT-PCR showed that when the concentration of EGS324 was 2.5 nM, the inhibition efficiency was 15%; and then 5 nM was 22%, 10 nM was 31%, 20 nM was 43%, 40 nM was 51%, 80 nM was 75%, and 160 nM was 75.6% (Fig. 5). The results showed that the inhibition efficiency of EGS324 was dose-dependent.

**Figure 6:**
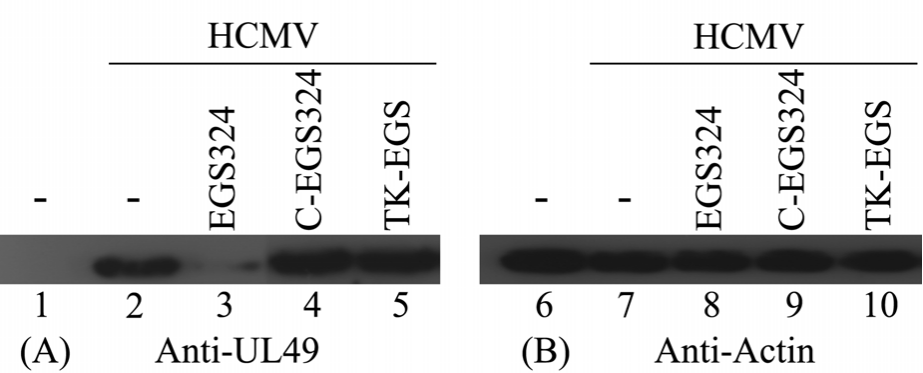
**In Western analyses (A&B), protein samples were separated in two identical SDS-polyacrylamide gels and transferred electrically to two identical membranes**. One membrane was allowed to react with a anti-ul49 antibody (A), whereas the other was stained with the monoclonal antibody (Anti-actin) against human actin (B).

The expression level of UL49 protein was also determined by Western analyses (Fig. 6). A reduction of 80% ± 3% in the expression level of UL49 protein was observed in cells treated with EGS324, whereas a reduction of less than 10% was found in the C-EGS324 treated cells.

### Inhibition of UL49 expression is not expected to affect the expression of other viral genes

It is possible that the observed reduction of UL49 expression in the EGS324-transfected cells is not necessarily due to specific EGS-directed RNase P cleavage of the target mRNA, but is due to other effects of the EGS on viral lytic replication, such as blocking the expression of viral immediate-early genes. To exclude these possibilities and further determine the antiviral mechanism of the EGS-directed cleavage, we examined the expression of other viral genes in the EGS324-treated cells. Relative RT-PCR analyses were carried out to determine the mRNA levels of an immediate-early gene (IE) and an early-late gene (UL44). Moreover, Western analyses were performed to determine the levels of viral protein IE2, a viral immediate-early (α) protein, gB, a viral early (β) protein, and pp28, a viral late (γ) protein. No significant differences in the expression levels of these genes were observed in cells that were treated with liposome complexes in the absence of EGS or in the presence of TK-EGS, EGS324, or C-EGS324 (Table 1). These results suggest that EGS324 specifically inhibits the expression of UL49 and does not affect overall viral gene expression.

**Table 1:** Inhibiting rates of the mRNA and protein expression of different viral genes in cells.

Viral genes	HFFs	EGS324	C-EGS324	TK-EGS
UL49 mRNA	0%	76% ± 3%	8%	3%
IE2 mRNA	0%	0%	0%	0%
UL44 mRNA	0%	0%	0%	0%
UL49 protein	0%	80% ± 3%	6%	5%
IE2 protein	0%	0%	0%	1%
Glycoprotein B	0%	2%	2%	1%
pp28 protein	0%	1%	2%	1%

The values shown are the means from triplicate experiments, and values of standard deviation that were less than 5% are not shown.

### Inhibition of HCMV growth in the EGS-treated cells

**Figure 7:**
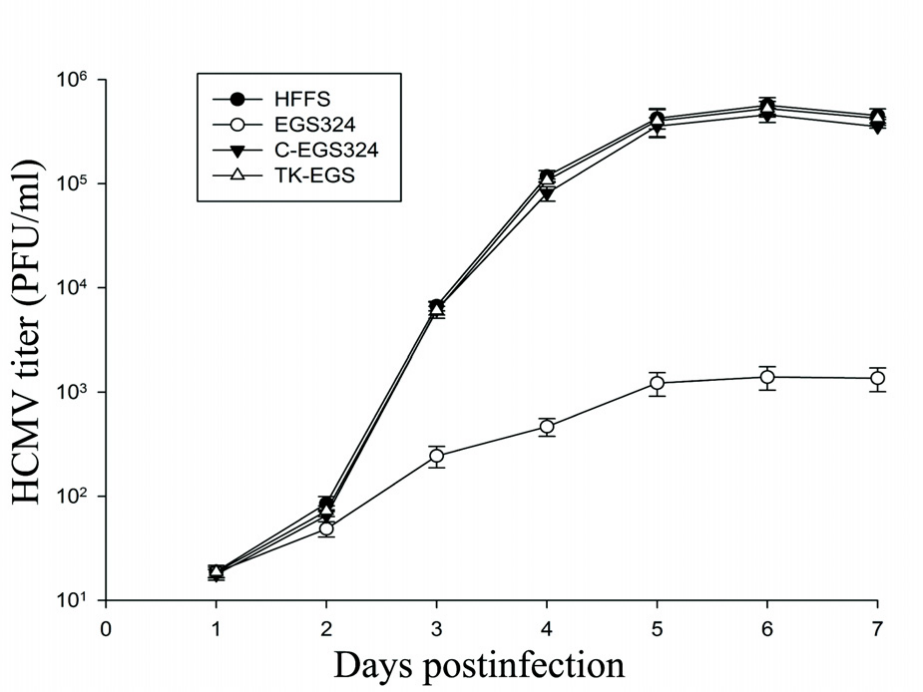
**Growth analysis of HCMV in human foreskin fibroblasts (HFFS) that were treated with liposome complexes in the absence of EGS, or in the presence of EGS324, C-EGS324 or TK-EGS**. 1 × 10^5 ^cells were first incubated with liposome complexes and then infected with HCMV at a moi of 1. Virus stocks were prepared from the infected cells at 1-day intervals through 7 days postinfection and viral titers were determined. The standard deviation is indicated by the error bars. These values are the means from triplicate experiments.

The impact of UL49 inhibition by EGS on viral growth was further investigated. Cells were treated with liposome complexes in the absence and presence of EGSs and then infected by HCMV at an moi of 1. Virus stocks were prepared from the infected cultures at 1-day intervals through 7 days postinfection. The count of plaque-forming units (PFU) was determined by measurement of the viral titer on cells. After 4 days postinfection, a reduction of about 330-fold in viral yield was observed in the EGS324-treated cells, whereas no significant reduction was found in those that were either treated with C-EGS324 or TK-EGS (Fig. 7). Thus, EGS324 is effective in inhibiting HCMV infection and blocking viral growth.

## Discussion

Compared to other nucleic acid-based gene interference approaches, the EGS technology with the use of endogenous human RNase P exhibits several unique and attractive features as a gene targeting tool. First, the mechanism of the EGS technology for degradation of a specific mRNA is different from other RNA- or DNA-based gene-targeting approaches. It uses the endogenous RNase P, which is one of the most ubiquitous, abundant, stable and efficient enzymes in all type of cells. This essential enzyme is highly expressed (5 × 10^4 ^copies per cell) and is responsible for the processing of all tRNA precursors that account for approximately 2% of total cellular RNA[24]. The action of RNase P with the EGS will result in irreversible cleavage of the target mRNA in a highly efficient catalytic fashion. Second, the sequence specificity of the EGS technology is governed by two different types of interactions between the EGS and the target mRNA: the base-pairing interactions in which the sequence of 12 nucleotides in the EGS hybridizes with the target mRNA, and the interactions between the target mRNA and the other part of the EGS sequence (equivalent to the T-stem and T-loop, and variable regions of a tRNA) which are required for folding of the RNase P-recognizable tertiary structure. Thus, the EGS-based technology is highly specific and does not generate nonspecific "irrelevant cleavage" that is observed in RNase H-mediated cleavage induced by conventional antisense phosphorothioate molecules [25].

Our study used exogenous administration of DNA-based EGSs for antiviral applications and demonstrated that RNase P-mediated targeting directed by DNA-based EGS was highly effective in inhibiting HCMV gene expression and viral growth in cultured cells. We showed that these EGS molecules directed human RNase P to cleave the UL49 mRNA sequence efficiently *in vitro*. Moreover, we also showed that these EGSs were readily delivered in cultured cells. A reduction of about 76~80% in the UL49 expression was achieved with a functional EGS, EGS324, whereas a reduction of less than 10% was observed in cells that were treated with C-EGS324 or TK-EGS. C-EGS324 bound to UL49 mRNA substrate *in vitro *as well as EGS324 but contained nucleotide mutations that disrupted RNase P recognition. These results suggested that the overall observed inhibition with EGS324 was primarily due to targeted cleavage by RNase P as opposed to the antisense effect or other nonspecific effects of the EGSs. Moreover, the antiviral effect of the EGS treatment (inhibition of viral growth) appeared to be due to the reduction of the UL49 expression. Only the expression of UL49 mRNA and protein was found to be reduced in cells treated with EGS324. We found no significant change in the expression of IE2, UL44, pp28, and gB (Table 1). The observed level of inhibition of UL49 expression was consistent with the extent of the reduction of viral growth.

HCMV is a member of the human herpesvirus family, which includes seven other different viruses such as HSV and Epstein-Barr virus. UL49 is well conserved and necessary for the growth of this virus [16]. Therefore, UL49 is considered as an ideal target for antiherpes therapy. To develop EGSs as a conventional drug that can be used as an exogenous agent for intracellular delivery in antiviral therapy, EGSs containing modified oligonucleotides are required to increase their stability *in vivo *[26,27]. It has been recently reported that chemically synthesized RNA-based EGSs with modified nucleotides can be administered exogenously into human cells and inhibit cellular gene expression [28]. Our study showed that exogenous administration of DNA-based EGSs is highly efficient in inhibiting gene expression and viral growth. Further understanding of how the functional groups in the nucleotides of an EGS interact with human RNase P and the mRNA substrate will lead to the construction of highly active and stable EGSs with either different bases or modifications at these nucleotide positions. Moreover, engineering different designs of EGSs [29] for increasing their targeting activity, as well as developing new means for improving their delivery, are needed to increase the efficacy of the EGSs *in vivo*. These studies will facilitate the development of the EGS-based technology for gene-targeting applications in both basic research and clinical therapy of HCMV infections.

## Competing interests

The authors declare that they have no competing interests.

## Authors' contributions

WJZ participated in gene cloning, sequence alignment, transfection, data analysis and drafting of the manuscript. YQL initially conceived of the study. ZFZ helped with PCR amplification on many of the sequences, XZ is responsible for the FQ-RT-PCR. SQL participated in western blotting. YZ revised the experiment design. THZ is the corresponding author. He assisted in the conception of data analyses, and in writing the manuscript. HJL is the supervising author. All authors read and approved the final manuscript.

## References

[B1] Mocarski ES Courcelle CT Knipe DM, Howley PM Cytomegaloviruses and their replication Fields virology 2001 Philadelphia PA: Lippincott-William & Wilkins 2629 2673

[B2] Baldanti F Lurain N Gerna G Clinical and biologic aspects of human cytomegalovirus resistance to antiviral drugs Human Immunology 2004 65 403 409 10.1016/j.humimm.2004.02.007 15172438

[B3] Stein CA Cheng Y Antisense oligonucleotides as therapeutic agents--is the bullet really magical? Science 1993 261 1004 1012 10.1126/science.8351515 8351515

[B4] Margraf S Bittoova M Vogel JU Kotchekov R Doerr HW Cinatl JJ Antisense oligonucleotide ISIS 2922 targets IE-expression and prevents HCMV-IE-induced suppression of TSP-1 and TSP-2 expression Nucleotides Nucleic Acids 2001 20 1425 1428 10.1081/NCN-100002569 11563036

[B5] Zhang G Raghavan B Kotur M Cheatham J Sedmak D Cook C Waldman J Trgovcich J Antisense transcription in the human cytomegalovirus transcriptome J Virol 2007 81 11267 11281 10.1128/JVI.00007-07 17686857 PMC2045512

[B6] Forster AC Altman S External guide sequences for an RNA enzyme Science 1990 249 783 786 10.1126/science.1697102 1697102

[B7] Werner M Rosa E Nordstrom JL Goldberg AR George ST Short oligonucleotides as external guide sequences for site-specific cleavage of RNA molecules with human RNase P RNA 1990 4 847 855 10.1017/S1355838298980323 PMC1369664 9671057

[B8] Zhang WJ Li HJ Li YQ He HQ Tang DS Zhang X Zhou TH Construction of an effective M1GS ribozyme targeting HCMV UL97 mRNA segment *in vitro* Yi Chuan Xue Bao 2005 32 1205 1212 16318287

[B9] Su YZ Li HJ Li YQ Chen HJ Tang DS Zhang X Jiang H Zhou TH *In vitro *construction of effective M1GS ribozymes targeting HCMV UL54 RNA segments Acta Biochim Biophys Sin 2005 37 210 214 15756425

[B10] Frank DN Pace NR Ribonuclease P: unity and diversity in a tRNA processing ribozyme Annu Rev Biochem 1998 67 153 180 10.1146/annurev.biochem.67.1.153 9759486

[B11] Yuan Y Hwang ES Altman S Targeted cleavage of mRNA by human RNase P Proc Natl Acad Sci USA 1992 89 8006 8010 10.1073/pnas.89.17.8006 1381505 PMC49844

[B12] Guerrier-Takada C Li Y Altman S Artificial regulation of gene expression in Escherichia coli by RNase P Proc Natl Acad Sci USA 1995 92 11115 11119 10.1073/pnas.92.24.11115 7479948 PMC40582

[B13] Kawa D Wang J Yuan Y Liu F Inhibition of viral gene expression by human ribonuclease P RNA 1998 4 1397 1406 10.1017/S1355838298980918 9814760 PMC1369712

[B14] Plehn-Dujowich D Altman S Effective inhibition of influenza virus production in cultured cells by external guide sequences and ribonuclease P Proc Natl Acad Sci USA 1998 95 7327 7332 10.1073/pnas.95.13.7327 9636148 PMC22606

[B15] Ma M Benimetskaya L Lebedeva I Dignam J Takle G Stein CA Intracellular mRNA cleavage induced through activation of RNase P by nuclease-resistant external guide sequences Nat Biotechnol 2000 18 58 61 10.1038/81113 10625392

[B16] Dunn W Chou C Li H Hai R Patterson D Stolc V Zhu H Functional profiling of a human cytomegalovirus genome Proc Natl Acad Sci USA 2003 100 14223 14228 10.1073/pnas.2334032100 14623981 PMC283573

[B17] Zhu J Trang P Kim K Zhou T Deng H Liu F Effective inhibition of Rta expression and lytic replication of Kaposi's sarcoma-associated herpesvirus by human RNase P Proc Natl Acad Sci USA 2004 101 9073 9078 10.1073/pnas.0403164101 15184661 PMC428475

[B18] Yang Y Li H Zhou T Kim K Liu F Engineered external guide sequences are highly effective in inducing RNase P for inhibition of gene expression and replication of human cytomegalovirus Nucleic Acids Res 2006 34 575 583 10.1093/nar/gkj431 16432261 PMC1345693

[B19] Trang P Lee M Nepomuceno E Kim J Zhu H Liu F Effective inhibition of human cytomegalovirus gene expression and replication by a ribozyme derived from the catalytic RNA subunit of RNase P from Escherichia coli Proc Natl Acad Sci USA 2000 97 5812 5817 10.1073/pnas.100101797 10811889 PMC18516

[B20] Zeng Z Li H Li Y Cui Y Zhou Q Zou Y Yang G Zhou T Effective inhibition of human cytomegalovirus gene expression by DNA-based external guide sequences Acta Biochim Biophys Sin 2009 41 389 398 10.1093/abbs/gmp024 19430703

[B21] Liu F Altman S Requirements for cleavage by a modified RNase P of a small model substrate Nucleic Acids Res 1996 24 2690 2096 10.1093/nar/24.14.2690 8758997 PMC145998

[B22] Yuan Y Altman S Selection of guide sequences that direct efficient cleavage of mRNA by human ribonuclease P Science 1994 263 1269 1273 10.1126/science.8122108 8122108

[B23] Altman S Kirsebom LA Gesteland RF Ribonuclease P The RNA world 1999 New York: Cold Spring Harbor Laboratory Press 351 380

[B24] Gopalan V Vioque A Altman S RNase P: variations and uses J Biol Chem 2002 277 6759 6762 10.1074/jbc.R100067200 11741968

[B25] Trang P Kim K Liu F Developing RNase P ribozymes for gene-targeting and antiviral therapy Cell Microbiol 2004 6 499 508 10.1111/j.1462-5822.2004.00398.x 15104592

[B26] Verma S Eckstein F Modified oligonucleotides: synthesis and strategy for users Annu Rev Biochem 1998 67 99 134 10.1146/annurev.biochem.67.1.99 9759484

[B27] Kim K Liu F Inhibition of gene expression in human cells using RNase P-derived ribozymes and external guide sequences Biochim Biophys Acta 2007 1769 603 612 17976837 10.1016/j.bbaexp.2007.09.001 PMC2705784

[B28] Li H Trang P Kim K Zhou T Liu F Effective inhibition of human cytomegalovirus gene expression and growth by intracellular expression of external guide sequence RNA RNA 2006 12 63 72 10.1261/rna.2184706 16301604 PMC1370886

[B29] Yang YH Li H Zhou T Kim K Liu F Engineered external guide sequences are highly effective in inducing RNase P for inhibition of gene expression and replication of human cytomegalovirus Nucleic Acids Res 2006 34 575 583 10.1093/nar/gkj431 16432261 PMC1345693

